# Pet caregiver burden in South Korea: key influences and the implications for veterinarians

**DOI:** 10.1186/s12917-025-04787-9

**Published:** 2025-05-09

**Authors:** Seola Joo, Hyomin Park, Myung-Sun Chun

**Affiliations:** 1 Research Institute for Veterinary Science, College of Veterinary Medicine, Seoul National University, Seoul, Republic of Korea; 2 Department of Urban Sociology, College of Urban Science, University of Seoul, Seoul, Republic of Korea

**Keywords:** Caregiver burden, Human-animal relationship, Veterinary care, Pet, Owner

## Abstract

**Background:**

The “caregiver burden” experienced by pet owners arises from the challenges and strains of caregiving, reflecting the complex relationship between humans and their pet animals and significantly impacting the quality of life of both humans and pets. This study aimed to quantitatively analyze the caregiver burden among South Korean pet owners and examine the various factors contributing to its formation.

**Methods and materials:**

A total of 766 dog and/or cat owners who identified themselves as primary caregivers were recruited through an online research panel. Participants completed questionnaires assessing pet and owner characteristics, including pet health status, attachment to the pet, caregiving support from family or others, and veterinary services. Statistical analyses, including regression analysis, were conducted using SPSS version 26.

**Results:**

The final regression model identified several factors associated with higher caregiver burden, including younger age (of both the pet and caregiver), caregiver gender (male), pet health status (acute or curable condition), level of support from others (lower emotional but higher financial and practical support), and higher frequency of veterinary communication for pet care. While strong attachment to pets may help alleviate caregiver burden, its effects are complicated and require careful consideration.

**Conclusions:**

Pet caregiver burden is shaped by a combination of pet-related, individual, and contextual factors. This study highlights the need for a relationship-centered approach to veterinary care that addresses the unique challenges faced by caregivers to enhance caregiver well-being and optimize pet welfare.

**Supplementary Information:**

The online version contains supplementary material available at 10.1186/s12917-025-04787-9.

## Background

South Korean society has seen a rapid increase in the number of households with pets over the past few decades, with pets now estimated to be present in about 15–20% of all households [1].^1^ With this shift, human-pet relationships have also been evolving, wherein pets are increasingly regarded as family members. Contributing factors include the extended lifespans of pets due to advancements in veterinary care and management, as well as growing awareness of health-related practices, all of which have led to a greater emphasis on “responsible ownership.” Taking responsibility for pets implies a commitment to providing adequate care throughout their lifetime, ensuring a certain quality of life (QoL).

Certain aspects of pet care are paralleled to human caregiving at home, as both aim to secure the health and well-being of the recipient. However, pet caregiving presents unique challenges. Pet owners often find themselves assuming new or expanded caregiving roles as they navigate through their pets’ different life stages, which is marked by a shorter lifespan and a complete dependency on human caregivers. This poses additional challenges, particularly when owners must make difficult end-of-life decisions, such as euthanasia [2, 3]. Goldberg [4] notes that in human healthcare, a range of support options is available when individuals require assistance, such as assisted living facilities, in-home health aides, and elderly care centers. However, pet owners often lack similar support systems, compelling them to serve as the equivalent of “assisted living facilities” for their pets by themselves. This responsibility can lead to feelings of guilt or frustration when owners struggle to meet caregiving demands, which may explain why pet care can sometimes feel overwhelming.

The idea that living with an animal can enhance owners’ QoL and have positive effects on health, psychological well-being, and social support is widely accepted [5–7]. However, empirical studies on the effects of pets on human health and well-being have yielded conflicting results [8]. This inconsistency is explained by the ‘pet-effect paradox’ [9], which partially links pet interactions to the burden of pet care through role-specific responsibilities. The “caregiver burden” [10] experienced by many pet owners, arising from the challenges and strains inherent in caregiving, highlights the complex relationship between humans and their companion animals. It encompasses both objective strains, such as time constraints, financial costs, and physical demands, as well as subjective emotional experiences [11]. Many pet-owning households view their pets as cherished family members and are committed to providing care throughout the pets’ lives. However, caregiver burden can have a profound impact on the QoL of both humans and animals [12]. For instance, excessive caregiver burden has been associated with increased stress levels and more pronounced symptoms of depression among pet owners [13, 14]. Research conducted by Krouzecky and colleagues [15] indicates that dog owners perceive daily stressors and challenges as more stressful than non-dog owners, suggesting that dog ownership may contribute to increased stress in everyday life. Additionally, a positive correlation was found between the strength of the human-dog bond and the perception of stressful life events, signifying that a stronger bond is associated with a heightened perception of stress during critical life events. Moreover, pet-related emotions—including feelings of anxiety, guilt, grief, depression, and poor psychological health that are often unrecognized, unacknowledged, or unsupported—may become disenfranchised when their meaning and experience are not understood by others, thereby invalidating pet owners’ practices [16].

The burdens associated with pet caregiving are closely linked to factors such as the pet’s health status, age, species, and specific care needs. Caregiving demands can vary by pet species; for instance, dogs typically require more attention due to their exercise and socialization needs, whereas cats are generally more independent [17]. Pets with medical conditions or behavioral challenges tend to create a greater caregiver burden, as they necessitate more intensive care, frequent veterinary visits, and often result in increased financial costs [2, 18]. The complexity of caregiving tasks can further intensify this burden [19]. Certain caregivers, particularly younger individuals [18] and those with limited financial resources [2, 20], may be more vulnerable to these challenges. Moreover, the level of social, emotional, and financial support from family or friends may correlate with caregiver burden [2, 13, 21].

The emotional attachment between pet owners and their pets can also have a significant impact on the caregiver’s experience of burden, producing contrasting outcomes. Stronger bonds may sometimes intensify stress, particularly when the pet is ill or nearing the end of life, as owners feel a deep sense of responsibility and emotional pain [3]. On the other hand, a strong human-animal bond can also serve as a source of emotional resilience, helping to alleviate caregiver burden by providing a sense of purpose [2]. This dual influence of attachment suggests that emotional connection can both increase the stress of caregiving and offer emotional support, depending on the context of the pet’s health and the caregiving demands.

The strain arising from caregiver burden can also extend to veterinarians, as distressed pet owners may channel their frustration into anger or complaints, a phenomenon referred to as “burden transfer” [22]. Such burden transfer can exacerbate occupational stress for veterinarians and negatively affect the veterinarian-client relationship, ultimately resulting in poorer treatment outcomes [4, 13, 23]. Furthermore, prior studies have identified financial concerns as the most significant burden faced by pet owners [2, 20]. Given that pet care—including medical procedures and compliance with legal requirements—occurs within a “veterinary regime” that encompasses social practices and institutional behaviors for pet owners [24, 25], access to financial options for veterinary services and resources can directly influence caregiving performance. Also, the quality of the veterinarian-client relationship—particularly the level of empathy and communication from veterinarians—affects the caregiver burden experienced by pet owners [19, 26, 27].

Based on this theoretical background, this study explores the complex, contextual, and reciprocal nature of pet caregiving by closely analyzing the challenges faced by pet owners in Korea, making it the first study to examine the caregiver burden experienced by Korean pet owners. Specifically, it investigates the various factors contributing to the formation of caregiver burden by examining a range of interconnected elements, including owner and pet characteristics, as well as the support for pet care from others and veterinary services. Furthermore, this study delves into the intricate relationship between pet caregiving and the emotional attachment to pets in shaping the overall experience of caregiver burden. By situating these findings within the Korean cultural context, this research provides a comprehensive understanding of the multifaceted dynamics involved in pet ownership and caregiver burden.

## Methods and materials

### Data collection and statistical analysis

Data collection took place over 19 days, from November 17 to December 5, 2022. Participants were recruited through the online panel managed by Ipsos, (Seoul, South Korea) a professional survey research company specializing in online data collection, utilizing population proportional allocation based on the 2020 Statistics Korea Census. This method ensured a representative sample across various cities and provinces, stratified by age group, household classification, and pet ownership status. The final sample included 766 participants, aged 20 to 69, who had dogs and/or cats as pets, and perceived themselves as a main caregiver who spent the most time and effort caring for the pets in their household. The data was analyzed using descriptive statistics, inferential tests such as t-tests, ANOVA and Pearson’s correlation coefficients, and regression analysis. All statistical analyses were executed using SPSS version 26. All study procedures were reviewed and approved by the Seoul National University Institutional Review Board (IRB No. 2112/001–002).

### Questionnaire

To examine the concerns addressed in the theoretical model, the questionnaire consists of four parts. The questionnaire is provided in Supplementary file I.

#### Part 1: participants and pets, and attachment to pets

The first section included questions about demographic factors such as age, gender, and household income. Participants were also asked to specify their household type (e.g., single adult, multiple adults, or households with children) as well as to identify their pet’s species (dog or cat), age, and perceived health status (healthy, curable symptoms and/or acute disease with a diagnosis, or chronic and/or terminal stage due to disease or aging). Health status was classified based on the duration and intensity of veterinary medical care required: “healthy status” referred to pets requiring no special medical care aside from preventive care; “curable and/or acute status” described pets needing short-term veterinary care with a relatively clear endpoint of treatment with the purpose of recovery; and “chronic and/or terminal status” applied to pets requiring continuous veterinary care or management to maintain their condition or slow deterioration. If participants had multiple pets, they were instructed to focus on the one that most frequently received veterinary treatment.

To measure the strength of participants’ attachment to their pets, the Lexington Attachment to Pets Scale (LAPS) was employed [28]. LAPS is widely used in research on human-animal relationships [29–31] and consists of 23 items rated on a 4-point Likert scale (0 = strongly disagree to 3 = strongly agree). Higher scores indicate stronger attachment between the owner and pet.

#### Part 2: support for pet care

Participants evaluated the support received from family and/or acquaintances across three categories [2, 3, 13]: Emotional support (e.g., “Do you feel that your family or acquaintances understand and empathize with your worries and challenges related to caring for your pet?”), financial support (e.g., “Do you receive financial support from family or acquaintances to help care for your pet?”), and practical support (e.g., “Do you receive practical assistance from family or acquaintances, such as shared caregiving or help when you’re away (e.g., at work, traveling, or on a business trip)?”). Responses were recorded on a 4-point Likert scale (0 = never, 1 = rarely, 2 = often, 3 = always), indicating the degree of support in each category.

#### Part 3: veterinary services

Participants described their experiences with veterinary services and communication with veterinarians. They provided information on the frequency of their annual veterinary visits and associated veterinary costs through open-ended responses. Recognizing that prior studies have identified financial concerns as a significant challenge for pet owners [2, 20], the survey also evaluated the perceived financial burden of veterinary costs using a 5-point Likert scale (1 = not burdensome at all to 5 = very burdensome, and 6 = No experience with veterinary clinic visits). Additionally, the survey assessed the frequency of socio-relational veterinary communication, including lifestyle-social topics (e.g., how does the pet get al.ong with the other members of the household? ) alongside biomedical information [32]. This socio-relational veterinary communication was examined across two dimensions: home care (e.g., medications, daily care routines) and life stage care (e.g., life-stage transitions like puppy/kitten stage, adulthood, and geriatric care and age-related concerns). These dimensions reflect a holistic approach, considering the animal’s well-being within the family context. Participants rated these dimensions using a 4-point Likert scale (0 = never, 1 = sometimes, 2 = every visit, 3 = often via call/email/messenger). The sum of these two scales was used as an indicator of the overall frequency of veterinary communication for pet care in subsequent regression analyses.

#### Part 4: caregiver burden

The final section of the survey evaluated the participants’ burden as pet caregivers. We employed a 7-item abbreviated version of the Zarit Burden Interview (ZBI), specifically adapted for pet owners [23, 33, 34]. The original ZBI was developed to measure the burden associated with caregiving, effectively capturing challenges related to functional impairments, behavioral issues, and contextual care demands, and has demonstrated strong internal consistency and validity [10, 35]. The abbreviated version [33] was designed to retain the psychometric integrity of the original scale while emphasizing the most prominent aspects of pet caregiver burden. Participants rated each item on a 5-point Likert scale (0 = never, 4 = nearly always), with higher scores on the 7-item ZBI indicating a greater perceived burden related to pet caregiving. The ZBI scale captures the subjective emotional toll of pet caregiving, focusing on stress, anger, and embarrassment resulting from the restrictions imposed by caregiving tasks. It also addresses negative emotional and psychological challenges, such as guilt and pressure stemming from perceived inadequacies in fulfilling caregiving duties, while excluding financial or other material aspects.

## Results

### Characteristics of participants and pets, and attachment to pets

Participants were comprised of 47.8% males and 52.2% females, with the majority aged over 50 years, accounting for 54.9% of the total. Additionally, 58.7% of participants reported a monthly household income exceeding 5,000,000 won (approximately 3,846 USD in 2022), which is significantly higher than the national average monthly household income of 3,718 USD.^2^ The most common family composition was that of two or more adults, accounting for 52.1% of participants, while single adult households represented only 8.6%. Families with children comprised 39.3% of participants (see Table 1).

Approximately 80% of the participants’ pets were dogs (*n* = 617) and 20% were cats. The average age of their pets was 5.4 years old. About 70% of the pets (*n* = 551) were regarded as healthy and free from any symptoms or diseases. In contrast, 16.8% exhibited curable symptoms or acute illnesses, while 11.2% were suffering from chronic or terminal conditions due to illness or aging. Additionally, 22.4% of the pets had other animals as companions within their households.

Participant’s attachment to their pet measured by LAPS showed a mean value of 50.91 (SD = 9.28). The score exhibited a group difference in gender (t = 4.418, *p* < 0.001, Cohen’s d = 9.177, 95% CI [0.177, 0.0.462]) where female caregivers indicated higher attachment. Though owner’s younger age (*r* = -0.072, *p* < 0.05, 95% CI [-0.142, -0.001]) showed a positive relation with high LAPS scores in the Pearson’s correlation analysis, the gap of mean difference was not significant among age groups according to ANOVA. When we analyzed participants’ household income (*r* = 0.100, *p* < 0.01, 95% CI [0.030, 0.170]) and pet age (*r* = 0.138, *p* < 0.05, 95% CI [0.068, 0.207]) as continuous variables, these factors had a significant positive correlation with LAPS. There were also significant mean differences among subgroups of household income (F_(3, 762)_ = 3.554, *p* < 0.05, η^2^ = 0.014, 95% CI [0.001, 0.031]) and those of pet age (F_(2, 763)_ = 3.747, *p* < 0.05, η^2^ = 0.010, 95% CI [0.000, 0.026]). Interestingly, participants who identified their pets to be in unhealthy status (F_(2, 763)_ = 8.323, *p* < 0.001, η^2^ = 0.021, 95% CI [0.005, 0.044]) showed higher attachment to their pets than those whose pets were perceived to be healthy. Also, participants who had more than two pets showed a statistically significant higher attachment score (t = -1.984, *p* < 0.05, Cohen’s d = 9.269, 95% CI [-0.332, -0.002]).

**Table 1:** Characteristics of participants and their pet

Characteristics			*N* (%)	LAPS	
				Mean (SD)	Quantitative tests
Owners	*Gender*	Male	366 (47.8)	49.37 (9.26) ^a^	t = 4.418 (*p* < 0.001)
		Female	400 (52.2)	52.31 (9.09) ^b^	
	*Age (year)*	20–29	50 (6.5)	53.34 (10.38)	F_(4, 761)_ = 1.915 (*p* = 0.106) *r* = -0.072 (*p* < 0.05)
		30–39	112 (14.6)	51.76 (8.76)	
		40–49	184 (24.0)	50.66 (9.18)	
		50–50	212 (27.7)	51.76 (8.55)	
		60<	208 (27.2)	49.80 (9.98)	
	*Household income (Monthly*,* 1*,*000won)*	< 3000	110 (14.1)	48.75 (10.48) ^a^	F_(3, 762)_ = 3.554 (*p* < 0.05) *r* = 0.100 (*p* < 0.01)
		3000–5000	206 (26.9)	50.25 (9.29) ^ab^	
		5000–8000	308 (40.2)	51.76 (8.92) ^b^	
		8000<	142 (18.5)	51.69 (8.81) ^b^	
	*Household type*	1 adult	66 (8.6)	48.75 (11.47)	F_(2, 763)_ = 2.418 (*p* = 0.090)
		More than 2 adults	399 (52.1)	50.81 (9.47)	
		Including kid(s)	301 (39.3)	51.50 (8.42)	
Pets	*Species*	Dog	617 (80.5)	50.78 (9.40)	t = 0.759 (*P* = 0.448)
		Cat	149 (19.5)	51.42 (8.79)	
	*Age (year)*	1–4	383 (40.0)	50.08 (8.60) ^a^	F_(2, 763)_ = 3.747 (*p* < 0.05) *r* = 0.138 (*p* < 0.01)
		5–9	268 (35.0)	51.37 (9.73) ^ab^	
		10≦	115 (15.0)	52.58 (10.16) ^b^	
	*Perceived health status*	Healthy	551 (71.9)	50.13 (9.24) ^a^	F_(2, 763)_ = 8.323 (*p* < 0.001)
		Acute or curable	129 (16.8)	52.05 (8.44) ^ab^	
		Chronic or terminal	86 (11.2)	54.16 (9.93) ^b^	
	*Number(s) of pet(s)*	1	579 (75.6)	50.53 (9.21) ^a^	t = -1.984 (*p* < 0.05)
		2≦	187 (22.4)	52.08 (9.43) ^b^	
Total			766 (100)	50.91 (9.28)	

LAPS = the Lexington Attachment to Pets Scale; Mean values sharing the same superscript letters (a, b) are not significantly different at *p* < 0.05 based on Tukey’s B test.

### Support for pet care

Support for pet care from family members and acquaintances varied across types. Emotional support was the most reported, with 88.8% of participants indicating they received it ‘often’ to ‘always.’ Financial support was reported by 52%, while 67.7% reported receiving practical support ‘often’ or ‘always’ (see Table 2).

**Table 2:** Support for pet care from family or acquaintances (%, (n))

Care support	Never	Rarely	Often	Always
Emotional support	0.4 (3)	10.8 (83)	79.5 (609)	9.3 (71)
Financial support	15.5 (119)	32.5 (249)	44.4 (340)	7.6 (58)
Practical support	8.2 (63)	24.2 (185)	58.4 (447)	9.3 (71)

### Veterinary services

**Fig. 1:**
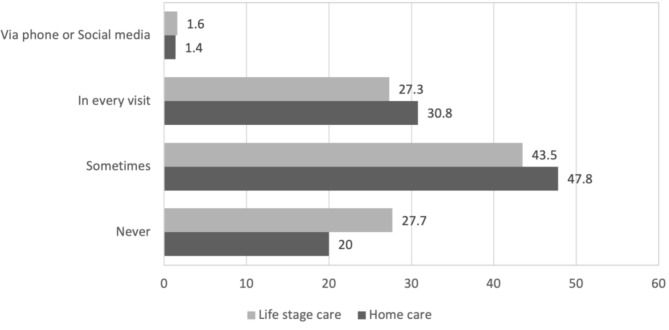
Frequency of communication on home and life stage care for pets with veterinarians (%, *n* = 766)

In response to questions regarding veterinary services, participants reported an average of 3.7 visits (SD = 3.8) to the veterinary clinic in the past year. 6.4% of participants had not visited a veterinary clinic during that period. Participants reported an average annual expenditure of approximately 527,000 won (approximately 405 USD) on veterinary medical care. As for financial burden from veterinary costs, a substantial proportion of participants (69.5%, *n* = 531) perceived veterinary medical costs in pet care expenditure as burdensome, with 17.9% describing them as ‘very burdensome’ and 51.6% as ‘burdensome.’ The average rating of the perceived burden from veterinary costs across all participants was 3.83 (SD = 0.76) on a five-point scale. There was a positive correlation between the perceived burden from veterinary costs and the actual expenditures, as indicated by Pearson’s correlation analysis (*r* = 0.194, *p* < 0.001, 95% CI [0.125, 0.262]). About one-third of participants engaged in communication about home care and life stage care for their pets during every veterinary visit. However, 20% had never engaged in discussions about home care practices and 27.7% (*n* = 212) about life stage care (see Fig. 1).

### Caregiver burden

Participants expressed a sense of burden regarding pet care, primarily because they felt they should be doing more for their pets and believed they could improve their caregiving (see Table 3). The average score for the ZBI was 11.80 (SD = 5.06), with scores ranging from 0 to 26, and a Cronbach’s alpha of 0.786. According to the cutoff reference values established by Spitznagel et al. [33], about two-thirds of participants fell into the normal (0–8, 27.8%, *n* = 213), mild (9–11, 23.6%, *n* = 181), and moderate (12–15, 25.5%, *n* = 195) burden categories, while 23.1% were classified as experiencing severe burden (16–26, *n* = 177).

**Table 3:** 7-item Zarit burden interview (*n* = 766)

7-item ZBI (Cronbach α = 0.786)	M (SD)
Do you feel that because of the time you spend with your pet that you don’t have enough time for yourself?	1.76 (1.09)
Do you feel stressed between caring for your pet and trying to meet other responsibilities for your family or work?	1.64 (1.13)
Do you feel you have lost control of your life since your pet’s illness?	1.56 (1.17)
Do you feel angry when you are around your pet?	0.79 (1.11)
Do you feel embarrassed over your pet’s behavior?	1.18 (1.15)
Do you feel you should be doing more for your pet?	2.23 (0.98)
Do you feel you could do a better job in caring for your pet?	2.64 (0.97)
Total	11.80 (5.06)

### Caregiver burden, perceived burden of veterinary costs, and actual cost of veterinary care for different pet health statuses

**Fig. 2:**
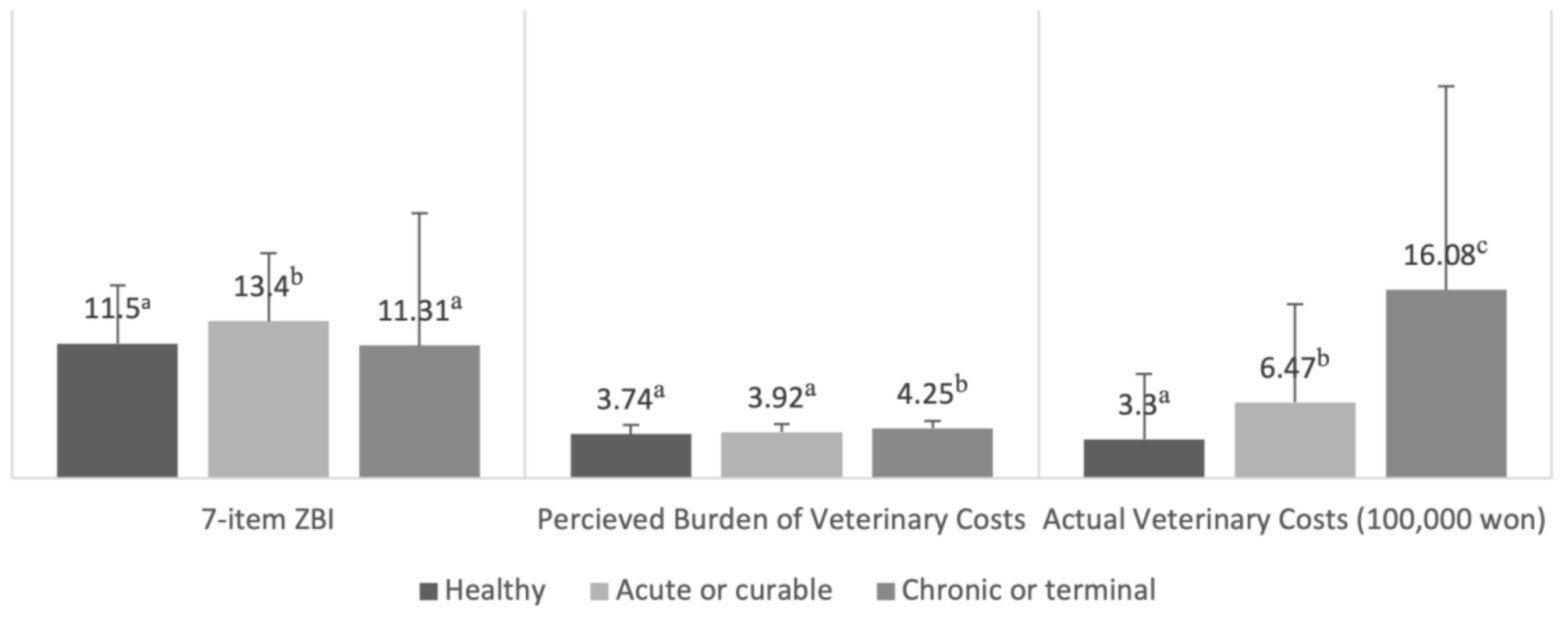
Group comparisons of Caregiver Burden (ZBI), Perceived Burden of Veterinary Costs, and Actual Veterinary Costs by pet health status groups (Mean values sharing the same superscript letters (a, b, c) are not significantly different at *p* < 0.05 based on Tukey’s B test.)

The analysis highlighted significant variances in caregiver burden, perceived burden of veterinary costs, and actual veterinary costs for different pet health status groups (see Fig. 2). Caregivers of pets with acute or curable conditions experienced the highest burden, with a mean ZBI score of 13.40 (SD = 5.79), which was significantly higher than those of healthy pets (M = 11.50, SD = 4.96) or pets with chronic/terminal illnesses (M = 11.31, SD = 4.01) (F_(2, 763)_ = 7.954, *p* < 0.001, η^2^ = 0.020, 95% CI [0.004, 0.043]). Additionally, the perceived financial burden of veterinary costs, rated on a five-point scale, differed significantly between pet health status groups (F_(2, 761)_ = 18.362, *p* < 0.001, η^2^ = 0.046, 95% CI [0.020, 0.077]). Caregivers of pets with chronic or terminal conditions reported higher perceived financial burden from veterinary costs (M = 4.25, SD = 0.63) than those of pets with acute or curable conditions (M = 3.92, SD = 0.68) and healthy pets (M = 3.74, SD = 0.77). Veterinary expenditures also varied significantly by pet health status, with respondents spending an average of 330,000 won (approximately 254 USD) per year for healthy pets, 647,000 won for pets with acute or curable conditions, and 1,608,000 won (approximately 1,237 USD) for pets with chronic or terminal conditions (F_(2, 763)_ = 91.016, *p* < 0.001, η^2^ = 0.193, 95% CI [0.144, 0.239]).

### Regression model of caregiver burden

We analyzed the associated factors to the burden of caring for pets using a hierarchical regression model with the caregiver burden (measured by the ZBI) as the dependent variable. The regression was developed in three models (see Table 4). Model 1 only included variables related to the owner (age, gender, and income), the pet (species, age, and health status), and human-animal relationship (attachment to pet; LAPS). Model 2 incorporated emotional, financial, and practical support from others as additional independent variables. Model 3 further included veterinary service factors, such as the frequency of veterinary visits per year, actual veterinary expenditures, and experience of communication with veterinarians about pet care.

Model 1 (F = 5.024, *p* < 0.001, adjusted R^2^ = 0.005) found the ages of the pet and caregiver (younger), the pet’s species (dog), the pet’s health status (acute and/or curable), and the level of attachment (lower) as significant predictors of pet caregiver burden. In Model 2, the inclusion of care support as variables revealed several significant predictors. Predictors identified by Model 1 (except for the species and attachment variables) continued to significantly contribute to the burden of care, but additionally, caregiver’s gender (male) emerged as a significant variable in Model 2. Also, low emotional support from others and, conversely, high economic and practical support, were found to be associated with a higher burden of care. The explanatory power of this model, which was statistically significant, accounted for approximately 13.4% of the variance in the burden of pet care (F = 11.742, *p* < 0.001, adjusted R^2^ = 0.134), representing an 7.8% improvement over Model 1. In Model 3, the inclusion of veterinary services as independent variables slightly increased the explanatory power to 14.1% (F = 9.990, *p* < 0.001, adjusted R^2^ = 0.141). In this model, higher frequency of communication with veterinarians about pet care was a significant factor explaining the burden of care, in addition to the predictors identified by Model 2 (except for practical care support).

**Table 4:** Regression model of pet caregiver burden (dependent variable = 7-item ZBI score)

Independent Variables	Model 1 (Pet-owner)			Model 2 (Care support)		Model 3 (Veterinary service)		
	β	t		β	t	β		t
(Constant)		11.663^***^			7.840^***^			8.005^***^
Owner gender (Male = 1)	0.050	1.386		0.071	2.042^*^	0.068		1.972^*^
Owner age	− 0.141	-3.937^***^		− 0.102	-2.953^**^	− 0.097		-2.820^**^
Household income	0.039	1.076		0.013	0.371	0.006		0.182
Species (Dog = 1)	0.074	2.079^*^		0.063	1.832	0.057		1.642
Pet age	− 0.111	-2.620^**^		− 0.101	-2.484^*^	− 0.096		-2.363^*^
Pet health status (Acute/curable = 1)	0.139	3.869^***^		0.123	3.571^***^	0.106		3.043^**^
Pet health status (Chronic/terminal = 1)	0.059	1.397		0.062	1.532	0.033		0.746
Attachment (LAPS)	− 0.093	-2.546^*^		− 0.047	-1.308	− 0.068		-1.828
Care support (Emotional)				− 0.120	-3.302^**^	− 0.131		-3.587^***^
Care support (Financial)				0.238	5.962^***^	0.237		5.932^***^
Care support (Practical)				0.089	2.262^*^	0.076		1.913
Veterinary visit(s) per year						0.042		0.962
Actual veterinary costs						0.030		0.686
Veterinary communication about pet care (Frequency)						0.089		2.534^*^
F	6.940^***^		11.742^***^				9.990^***^	
Adjusted R^2^	0.058		0.134				0.141	

β = standardized coefficient, LAPS = the Lexington Attachment to Pets Scale, ^*^p < 0.05, ^**^p < 0.01, ^***^p < 0.001

## Discussion

This study aimed to investigate the caregiver burden experienced by pet owners in South Korea using the ZBI. The findings offer a comprehensive understanding of the many-sided factors contributing to the perceived caregiving burden, encompassing pet and owner characteristics, the support for pet care from others, and veterinary services within the South Korean context.

### Pet caregiver burden represented on the ZBI

According to Spitznagel et al. [33], reference values from large-scale studies in the U.S. suggest that a ‘normal’ caregiver burden for healthy pets typically ranges from 0 to 8 points on the ZBI. However, caregivers of healthy pets in our study reported higher burdens (M = 11.50, SD = 4.9) compared to these reference values. Several cultural and societal factors in South Korea may have contributed to this phenomenon. The first is the high expectation of what constitutes responsible pet ownership. In recent years, there has been a growing emphasis in Korea on the ethical and responsible treatment of pets, alongside legal reinforcements of pet owners’ obligations, and numerous media reports dealing with pet owners’ duty of care. This social expectation or shift toward treating pets as family cause caregivers to feel heightened pressure to meet higher standards of care. It leads to a disproportionately increased burden on owners who strive to meet these evolving expectations [36]. Secondly, the long working hours and demanding workloads in South Korea hinder caregivers from properly attending to their pets. Healthy pets still require significant emotional and physical engagement, which can be challenging for caregivers with busy lifestyles. The stress of being unable to spend sufficient time with pets or adequately meet their needs due to work or social commitments can amplify the perceived burden, particularly if owners feel guilt or anxiety about neglecting their pets [16]. The growing need for and use of pet care support systems, such as dog daycare centers or pet hotels, in South Korea can be linked to pet owners’ perceived burden from leaving their pets alone or being unable to care for them while at work.

On the other hand, caregivers of pets with acute and/or curable conditions experienced a higher burden compared to those of healthy pets or those with chronic and/or terminal conditions. This heightened burden likely stems from the emotional stress associated with the lack of predictability [13, 27]. Acute and/or curable conditions can be unpredictable, often requiring urgent veterinary visits, new medication administration, and short-term but intensive care, all of which can disrupt the caregiver’s routine and add stress [23]. For pets with chronic or terminal conditions, caregivers can adjust to the long-term nature of care and develop coping strategies over time. Spitznagel et al. [37] also found that when a disease is well managed, the burden on caregivers tends to diminish. Moreover, while chronic and/or terminal conditions often come with a degree of acceptance of illness, there is usually a clear path to recovery for acute conditions which lends to a sense of urgency and immediate responsibility. The expectation of recovery may also lead to greater self-imposed pressure to ‘do everything possible’ for the pet, thereby further intensifying the perceived burden. Although there was no statistical difference in caregiver burden between the chronic and/or terminal group and the healthy group, the chronic and/or terminal group faced the highest financial burden among the three groups, likely due to rising medical expenses.

Findings from this research also suggest that the perceived care burden interplays with the owner’s and pet’s life stages. We found that caregiver burden intensifies with younger pets, due to the shorter duration of time the owners and pets have spent together. This pattern aligns with prior research suggesting that the bond between pet owners and their animals strengthens over time, often accompanied by improved perceptions of pet behavior and reduced caregiving challenges. A recent study examining pet owners’ perceptions and understanding of their dogs’ behavior found that respondents’ positive feelings toward their dogs’ behavior gradually improved over time as pets often become calmer and less demanding as they mature, which helps strengthen the bond between pet owners and their pets [17]. The owner’s perception and sense of responsibility regarding their pet’s behavior and changes can significantly influence daily care routines, which, in turn, affect both the pet’s welfare and the owner’s overall well-being. This finding underscores the nature of caregiving as a dynamic and evolving process, rooted in experiential knowledge and sustained through relational, habitual, and accumulated understanding [38, 39]. As for owner factors, participants of younger ages felt a higher pet caregiver burden. As Graham and colleagues [40] point out, young pet owners (millennials) often rely on others, such as parents, roommates, and partners, to help them care for their pets. This reliance was often due to lifestyle constraints, limited resources, or financial challenges, such as paying for surgery or managing care while they were at school or work. These factors can create difficulties and serve as a significant source of stress for younger pet owners.

Our research outcomes reveal that attachment to pets plays a significant role in alleviating caregiver burden when considering only the factors related to pet and owner characteristics. Interestingly, the results showed that the pet’s health status does not weaken attachment levels. Rather, a pet’s poor health often motivates caregivers to seek solutions and invest more in care to enhance their pet’s comfort. Consistent with the previous study suggested by Belshaw, Dean, and Asher [41], this bond reduces the perceived burden of caregiving on lifestyle changes. High levels of caregiving are frequently associated with strong attachment [42], and caregiving activities, in turn, promote attachment development, creating a positive feedback loop that sustains caregiving efforts [2]. However, when we incorporated support and veterinary service factors into our regression analysis, the influence of attachment on caregiver burden disappeared. This suggests that the caregiving environment may have a stronger impact on burden than attachment alone, which again underscores the intricate relationship between attachment and caregiver burden. While attachment can alleviate emotional distress and strengthen caregiving motivation, it may also contribute to financial and practical stress. Studies indicate that higher attachment levels correlate with increased veterinary expenses and financial burden [21, 43, 44]. Hence, the effects of attachment on caregiving burden are complicated and should be interpreted with caution.

It is noteworthy that male caregivers report a higher burden of pet care in the regression models (2 and 3) than female caregivers. Several perspectives offer the interpretation of this finding, particularly concerning the challenges faced by male primary caregivers. First, social expectations and traditional gender roles in South Korea can influence the different caregiving patterns between genders. Traditionally, men in South Korea have mainly been responsible for breadwinning roles rather than caregiving roles and have felt less social pressure to care for family or relatives [45]. When men assume the primary caregiving role for pets, they may experience stress due to a dissonance between existing social norms and their role in the family. Second, a lack of experience as a caregiver can aggravate the burden. Men with limited experiences with caregiving may find it challenging to manage the routine and specific demands of pet care, such as addressing health needs, cleaning, and providing emotional support, all of which require significant time and effort. Third, emotional connection and psychological burden are important considerations. Male caregivers may feel an overwhelming sense of restriction stemming from pet care, compounded by relatively lower levels of attachment compared to female caregivers. This disparity can heighten stress, particularly when faced with health problems or behavioral issues. Furthermore, men often deal with their negative emotions independently rather than sharing them and seeking social support [46], which may exacerbate their caregiving burden. Similar patterns have been observed in caregiving contexts for people. For example, male caregivers—many of whom are single—dedicated to caring for aging parents reported the highest-burden among all caregivers [47]. While comparable to women in their sense of filial duty, these men often experienced emotional isolation as they navigated caregiving responsibilities within the constraints of social expectations around masculinity. To the best of our knowledge, no studies have directly examined gender differences in pet caregiver burden yet. Thus, future research should explore the unique challenges faced by male caregivers, shedding light on their experiences to better address their needs and reduce their burden.

Our findings highlight the importance of support networks—emotional, financial, and practical—in influencing caregiver burden. Consistent with previous studies [14, 18], the lack of emotional support, such as not having someone to talk to about pet care, was strongly associated with increased caregiver burden. Emotional support has been shown to have a statistically significant effect in alleviating stress, emphasizing the importance of social connections in reducing caregiver burden. Interestingly, our results revealed that financial and practical support are positively associated with caregiver burden. It appears counterintuitive and inconsistent with prior studies. Spitznagel et al. [18] suggest that tangible and practical support—like having someone assist with caregiving tasks—can alleviate caregiver burden, especially when dealing with pets experiencing severe health issues. Similarly, Britton [2] argued that feelings of inadequacy in caregiving can arise from challenges such as time constraints, caregiving difficulties, and a lack of supportive resources, often resulting in suboptimal care and heightened guilt. However, our findings suggest that the need for financial and practical support may reflect situations necessitating intensive caregiving, which leads to a greater overall burden. For example, caregivers requiring more assistance may already be managing tough caregiving scenarios, including pets with acute or chronic health conditions. This aligns with the idea that the presence of external support can act as a marker of high caregiving demands rather than a purely alleviating factor. Consequently, while such support may partially ease the strain, it also underscores the elevated level of care required, contributing to caregivers’ stress. Additionally, cultural norms in South Korea, including face-saving and collectivist values, may discourage seeking help due to the social expectation of avoiding inconvenience to others. However, self-reliance is emphasized in many societies, suggesting that individual and psychological factors also play a role. While Korean culture may reinforce this tendency, attributing it solely to cultural influences would be an oversimplification. Collectively, these findings point to the critical role of the surrounding environment in shaping caregiving experiences. Emotional, financial, and practical supports significantly impact the quality of care provided to pets and the overall well-being of caregivers, highlighting the multifaceted dynamics of caregiving systems.

In a similar vein, while neither the frequency of veterinary visits nor veterinary-associated expenses directly affect caregiver burden, the frequency of communication with veterinarians about pet care was positively associated with participants’ sense of burden (*p* < 0.001). It reflects the situations where heightened caregiver burden leads to increased reliance on support factors. This is inferred as “burden transfer,” suggesting that caregivers experiencing stress are more inclined to seek reassurance and support from veterinary professionals [22]. Distressed caregivers, often anxious and overwhelmed, may frequently engage in nonbillable interactions, seeking emotional reassurance. However, this behavior can unintentionally shift stress onto veterinarians, sometimes manifesting as client dissatisfaction or complaints, which in turn may negatively impact veterinarians’ occupational well-being. The findings call attention to the need for strategies to address and mitigate caregiver burden within veterinary contexts.

### Implications for veterinarians

Caregiver burden encompasses the emotional, practical, and relational challenges of pet care. Addressing these challenges is essential for providing adequate support for both caregivers and their pets. However, veterinary consultations often emphasize only on biomedical issues, leaving caregivers without guidance on managing caregiving challenges [32]. Veterinarians can play a pivotal role by incorporating tailored advice throughout a pet’s life stages, addressing lifestyle and social factors and fostering strong Veterinary-Client-Patient Relationship (VCPR). Initiating conversations with clients about their caregiving burden and discussing their experiences may help alleviate stress and better address the unique challenges faced by pet owners [33, 48].

Previous studies reported a strong link between caregiver burden and veterinary communication. Effective communication can alleviate caregiver stress, while communication issues—especially around costs—can intensify it [26, 49]. Burden often arises from managing complex medication regimens or disruptive treatment schedules [13]. Misunderstandings about costs may further reduce compliance with treatment recommendations, negatively impacting outcomes [4, 23]. Veterinarians can help alleviate caregiver burden by adopting a relationship-centered approach to care, acknowledging the complexities of the human-animal bond. The “contextualized care” model, which emphasizes individualized and realistic care plans over rigid adherence to “gold-standard” ideals, can help reduce feelings of guilt, shame, or dissatisfaction among caregivers and veterinary teams [50]. Empathy and clear communication are critical, particularly when navigating complex treatments or discussing costs, as miscommunication in these areas often heightens caregiver stress [26].

While compassionate communication skills are of utmost importance in ensuring the quality of services provided to pets, limited attention is given to training veterinarians in this area. Veterinary education in communication skills, compassion, and rapport-building remains insufficient, as these are often regarded as secondary skills implicitly acquired through the hidden curriculum. A structured course on these competencies should be integrated into the formal veterinary curriculum alongside biomedical and technical training to better prepare veterinarians for professional practice [51, 52].

### Limitations and further directions

This study is the first in South Korea to examine the burden experienced by pet owners; still, it has certain limitations. The participants may not fully represent the general population of pet owners and may not reflect experiences in situations involving care failures or critical challenges. Future studies will benefit from examining pet owners’ caregiving burden in samples representing various populations, such as those reflecting different practice types (e.g., farm settings, production animals, general clinics, referral clinics, or animal cancer centers) or certain disease groups. For clarity in data collection and analysis, we limited our sample to dog and cat owners, as these are the most commonly kept pets in South Korea, rather than including all pet types. However, caregiver burden is likely to vary by species due to differences in size, physical and behavioral characteristics, dietary requirements, level of care, living environment, and the nature of human-animal interactions. Future research should explore the burden associated with caring for other types of pets, such as birds, reptiles, and other small mammals, to provide comparative insights. Further research is also needed on veterinarians’ perspectives regarding pet caregiver burden to develop a more holistic understanding of the concept and to enhance communication between veterinarians and pet owners. Another critical area for future research is to examine more fully whether and how client caregiver burden impacts veterinary staff workload and occupational stress in veterinarians. If such associations exist, interventions to decrease client caregiver burden should be assessed for downstream effects on work-related stress in veterinarians [22]. Cross-country comparative studies may also deliver interesting insights into how cultural, economic, and social factors influence the caregiver burden of pet owners. Additionally, longitudinal studies could provide insights into how shifting societal norms and work-life dynamics impact pet care burdens in South Korea. These efforts will be instrumental in improving the quality of care for pets and their owners while addressing the broader implications for veterinary professionals.

## Conclusion

As the primary investigation into South Korean pet owners’ caregiver burdens, this study offers critical insights into the factors contributing to such burdens. The findings revealed that pet caregiver burdens are influenced by a combination of pet-related, individual, and contextual factors. Specifically, younger pets and shorter durations of time spent together were associated with higher levels of burden, highlighting the challenges of developing caregiving routines early in the caregiving journey. Caregivers of pets with acute or curable conditions reported the highest levels of burden, likely due to the demands of managing unpredictable and challenging situations. Younger and male caregivers experienced greater burdens, highlighting the critical need for targeted support for these groups. Emotional support emerged as a critical mitigating factor, while a lack thereof significantly increased caregiver burden. Conversely, more financial and practical support from family and/or others and higher frequency of veterinary communication about pet care were linked to a higher burden, likely reflecting the existence of heavily-intensive demands in pet care scenarios. While strong attachment to pets may help alleviate caregiver burden, its effects are complicated and require careful consideration.

From a veterinary perspective, this study underscores the importance of adopting a relationship-centered approach to care. Empathetic communication and contextualized care strategies are essential for supporting pet owners. By fostering strong veterinary-client relationships and addressing the unique challenges faced by caregivers, veterinarians can enhance caregiver well-being and optimize pet welfare.

## Electronic supplementary material

Below is the link to the electronic supplementary material.

Supplementary Material 1

## Data Availability

The datasets used and/or analyzed during the current study are available from the corresponding author on reasonable request.

## Footnotes

1 In 2020, the National Census of Population and Housing, conducted every five years, included a survey item on pet ownership for the first time. As the largest-scale survey in Korea, the census covered approximately 20% of all households nationwide. The results estimate that 3.129 million households, or about 15% of the total, have pets. Among these, 2.423 million households (11.6%) have dogs, while 717,000 households (3.4%) have cats.

2 Korean Statistical Information Service. [cited 2025 Feb 10]. Available from: https://kosis.kr/eng/.

## Abbreviations

ZBI: The Zarit Burden Interview
QoL: Quality of life
LAPS: The Lexington Attachment to Pets Scale
VCPR: Veterinary-Client-Patient Relationship

## References

[1] Statistics Korea. Population and housing census sample count results. Statistics Korea Press Release; 2020.

[2] Britton K, Galioto R, Tremont G, Chapman K, Hogue O, Carlson MD, et al. Caregiving for a companion animal compared to a family member: burden and positive experiences in caregivers. Front Vet Sci. 2018;5(325). 10.3389/fvets.2018.00325. 10.3389/fvets.2018.00325 PMC6308119 30619903

[3] Christiansen SB, Kristensen AT, Sandøe P, Lassen J. Looking after chronically ill dogs: impacts on the caregiver’s life. Anthrozoös. 2013;26(4):519–33. 10.2752/175303713X13795775536174.

[4] Goldberg KJ. Exploring caregiver burden within a veterinary setting. Vet Rec. 2017;181(12):318–9. 10.1136/vr.j4156. 28923851 10.1136/vr.j4156

[5] Allen K. Are pets a healthy pleasure? The influence of pets on blood pressure. Curr Dir Psychol Sci. 2003;12(6):236–9.

[6] Stanley IH, Conwell Y, Bowen C, Van Orden KA. Pet ownership May attenuate loneliness among older adult primary care patients who live alone. Aging Ment Health. 2014;18(3):394–9. 24047314 10.1080/13607863.2013.837147 PMC3944143

[7] Carr EC, Wallace JE, Pater R, Gross DP. Evaluating the relationship between well-being and living with a dog for people with chronic low back pain: A feasibility study. Int J Environ Res Public Health. 2019;16(8):1472. 31027281 10.3390/ijerph16081472 PMC6517939

[8] Herzog H. The impact of pets on human health and psychological well-being: fact, fiction, or hypothesis? Curr Dir Psychol Sci. 2011;20(4):236–9.

[9] Krouzecky C, Aden J, Hametner K, Klaps A, Kovacovsky Z, Stetina BU. Fantastic Beasts and Why It Is Necessary to Understand Our Relationship—Animal Companionship under Challenging Circumstances Using the Example of Long-Covid. Animals. 2022;12(15):1892. Available from: https://www.mdpi.com/2076-2615/12/15/1892 10.3390/ani12151892 PMC9330774 35892542

[10] Zarit SH, Reever KE, Bach-Peterson J. Relatives of the impaired elderly: correlates of feelings of burden. Gerontologist. 1980;20(6):649–55. 10.1093/geront/20.6.649. 7203086 10.1093/geront/20.6.649

[11] Spitznagel MB, Anderson JR, Marchitelli B, Sislak MD, Bibbo J, Carlson MD. Owner quality of life, caregiver burden and anticipatory grief: how they differ, why it matters. Vet Rec. 2021;188(9):e74. 33960467 10.1002/vetr.74

[12] Kogan LR, Wallace JE, Hellyer PW, Carr EC. Canine caregivers: Paradoxical challenges and rewards. Animals. 2022a;12(9):1074. 35565501 10.3390/ani12091074 PMC9099636

[13] Shaevitz MH, Tullius JA, Callahan RT, Fulkerson CM, Spitznagel MB. Early caregiver burden in owners of pets with suspected cancer: owner psychosocial outcomes, communication behavior, and treatment factors. J Vet Intern Med. 2020;34(6):2636–44. 32969546 10.1111/jvim.15905 PMC7694845

[14] Spitznagel MB, Carlson MD. Caregiver burden and veterinary client well-being. Vet Clin Small Anim Pract. 2019a;49(3):431–44. 10.1016/j.cvsm.2019.01.008 30846380

[15] Krouzecky C, Emmett L, Klaps A, Aden J, Bunina A, Stetina BU. And in the middle of my chaos there was you?—Dog companionship and its impact on the assessment of stressful situations. Int J Environ Res Public Health. 2019;16(19):3664. 31569522 10.3390/ijerph16193664 PMC6801798

[16] Kogan LR, Bussolari C, Currin-McCulloch J, Packman W, Erdman P. Disenfranchised guilt—Pet owners’ burden. Animals. 2022b;12(13):1690. 35804588 10.3390/ani12131690 PMC9264879

[17] Owczarczak-Garstecka SC, Da Costa REP, Harvey ND, Giragosian K, Kinsman RH, Casey RA et al. It’s Like Living with a Sassy Teenager! A Mixed-Methods Analysis of Owners’ Comments about Dogs between the Ages of 12 Weeks and 2 Years. Animals. 2023;13(11):1863. Available from: https://www.mdpi.com/2076-2615/13/11/1863 10.3390/ani13111863 PMC10252082 37889782

[18] Spitznagel MB, Jacobson DM, Cox MD, Carlson MD. Predicting caregiver burden in general veterinary clients: contribution of companion animal clinical signs and problem behaviors. Vet J. 2018;236:23–30. 10.1016/j.tvjl.2018.04.007. 29871745 10.1016/j.tvjl.2018.04.007

[19] Spitznagel MB, Patrick K, Hillier A, Gober M, Carlson MD. Caregiver burden, treatment complexity, and the veterinarian–client relationship in owners of dogs with skin disease. Vet Dermatol. 2022;33(3):208–13. 35293042 10.1111/vde.13065 PMC9311805

[20] Meier C, Maurer J. Buddy or burden? Patterns, perceptions, and experiences of pet ownership among older adults in Switzerland. Eur J Ageing. 2022;19. 10.1007/s10433-022-00696-0. 10.1007/s10433-022-00696-0 PMC9729639 36506656

[21] Corr SA, Lund TB, Sandøe P, Springer S. Cat and dog owners’ expectations and attitudes towards advanced veterinary care (AVC) in the UK, Austria and Denmark. PLoS ONE. 2024;19(3):e0299315. 38507341 10.1371/journal.pone.0299315 PMC10954172

[22] Spitznagel MB, Ben-Porath YS, Rishniw M, Kogan LR, Carlson MD. Development and validation of a burden transfer inventory for predicting veterinarian stress related to client behavior. J Am Vet Med Assoc. 2019b;254(1):133–44. 30668296 10.2460/javma.254.1.133

[23] Spitznagel MB, Jacobson DM, Cox MD, Carlson MD. Caregiver burden in owners of a sick companion animal: a cross-sectional observational study. Vet Rec. 2017;181(12):321. 10.1136/vr.104295. 28870976 10.1136/vr.104295

[24] Swabe J. Animals, disease and human society: human-animal relations and the rise of veterinary medicine. Routledge; 1999..p8.

[25] Soares JBPC. Veterinary Anthropology and Companion Species: technoscience, pet owners and ethics of care. 2020.

[26] Pilgram MD. Communicating social support to grieving clients: the veterinarians’ view. Death Stud. 2010;34(8):699–714. 24482846 10.1080/07481181003761666

[27] Christiansen SB, Kristensen AT, Lassen J, Sandøe P. Veterinarians’ role in clients’ decision-making regarding seriously ill companion animal patients. Acta Vet Scand. 2016;58(1):1–14. 27221809 10.1186/s13028-016-0211-x PMC4879734

[28] Johnson TP, Garrity TF, Stallones L. Psychometric evaluation of the Lexington attachment to pets scale (LAPS). Anthrozoös. 1992;5(3):160–75.

[29] Wells DL, Clements MA, Elliott LJ, Meehan ES, Montgomery CJ, Williams GA. Quality of the Human–Animal bond and mental wellbeing during a COVID-19 lockdown. Anthrozoös. 2022;1:20.

[30] Meehan M, Massavelli B, Pachana N. Using attachment theory and social support theory to examine and measure pets as sources of social support and attachment figures. Anthrozoös. 2017;30(2):273–89.

[31] Shore ER, Douglas DK, Riley ML. What’s in it for the companion animal? Pet attachment and college students’ behaviors toward pets. J Appl Anim Welf Sci. 2005;8(1):1–11. 16004541 10.1207/s15327604jaws0801_1

[32] Shaw JR, Bonnett BN, Adams CL, Roter DL. Veterinarian-client-patient communication patterns used during clinical appointments in companion animal practice. J Am Vet Med Assoc. 2006;228(5):714–21. 16506932 10.2460/javma.228.5.714

[33] Spitznagel MB, Mueller MK, Fraychak T, Hoffman AM, Carlson MD. Validation of an abbreviated instrument to assess veterinary client caregiver burden. J Vet Intern Med. 2019c;33(3):1251–9. 31033026 10.1111/jvim.15508 PMC6524077

[34] Leonardi AJ, Fulkerson CM, Shields CG, Childress MO. Veterinary oncologists and pet owners differ in their perceptions of chemotherapy-related adverse events in cancer-bearing dogs. J Am Vet Med Assoc. 2024;262(3):334–42.38041950

[35] Ankri J, Andrieu S, Beaufils B, Grand A, Henrard JC. Beyond the global score of the Zarit burden interview: useful dimensions for clinicians. Int J Geriatr Psychiatry. 2005;20(3):254–60. 15717336 10.1002/gps.1275

[36] Westgarth C, Christley RM, Marvin G, Perkins E. The responsible dog owner: the construction of responsibility. Anthrozoös. 2019;32(5):631–46. 10.1080/08927936.2019.1645506.

[37] Spitznagel MB, Solc M, Chapman KR, Updegraff J, Albers AL, Carlson MD. Caregiver burden in the veterinary dermatology client: comparison to healthy controls and relationship to quality of life. Vet Dermatol. 2019d;30(1):3–e2. 30370700 10.1111/vde.12696

[38] de La Bellacasa MP. Matters of care: speculative ethics in more than human worlds. Volume 41. U of Minnesota; 2017.

[39] Hamington M, Care. Moral progress, and companion animals. Pets and people: the ethics of our relationships with companion animals. Oxford University Press; 2017. pp. 49–63.

[40] Graham TM, Milaney KJ, Adams CL, Rock MJ. Are millennials really picking pets over people? Taking a closer look at dog ownership in emerging adulthood. Can J Fam Youth. 2019;11(1):202–27.

[41] Belshaw Z, Dean R, Asher L. You can be blind because of loving them so much: the impact on owners in the united Kingdom of living with a dog with osteoarthritis. BMC Vet Res. 2020;16(1):190. 32527313 10.1186/s12917-020-02404-5 PMC7291569

[42] Kurdek LA. Pet dogs as attachment figures. J Soc Pers Relatsh. 2008;25(2):247–66. Available from: 10.1177/0265407507087958

[43] Williams A, Williams B, Hansen CR, Coble KH. The impact of pet health insurance on dog owners’ spending for veterinary services. Animals. 2020;10(7):1162. 32659934 10.3390/ani10071162 PMC7401533

[44] Quain A, Ward MP, Mullan S. Ethical challenges posed by advanced veterinary care in companion animal veterinary practice. Animals. 2021;11(11):3010. 34827742 10.3390/ani11113010 PMC8614270

[45] Campbell LD. Sons who care: examining the experience and meaning of filial caregiving for married and Never-Married sons. Can J Aging / La Revue Canadienne Du Vieillissement. 2010;29(1):73–84. 10.1017/S071498080999033X 20202266

[46] Sanders S, Power J. Roles, responsibilities, and relationships among older husbands caring for wives with progressive dementia and other chronic conditions. Health Soc Work. 2009;34(1):41–51. 19281101 10.1093/hsw/34.1.41

[47] Friedemann ML, Buckwalter KC. Family caregiver role and burden related to gender and family relationships. J Fam Nurs. 2014;20(3):313–36. 10.1177/1074840714532715. 24777069 10.1177/1074840714532715 PMC4442741

[48] Spitznagel MB, Marchitelli B, Gardner M, Carlson MD. Euthanasia from the veterinary client’s perspective: psychosocial contributors to euthanasia decision making veterinary clinics of North America. Small Anim Pract. 2020;50(3):591–605. 10.1016/j.cvsm.2019.12.008 32115280

[49] Pasteur K, Diana A, Yatcilla JK, Barnard S, Croney CC. Access to veterinary care: evaluating working definitions, barriers, and implications for animal welfare. Front Vet Sci. 2024;11. 10.3389/fvets.2024.1335410 PMC10830634 38304544

[50] Skipper A, Gray C, Serlin R, O’Neill D, Elwood C, Davidson J. Gold standard care’ is an unhelpful term. Vet Rec. 2021;189(8):331. 10.1002/vetr.1113. 34677842 10.1002/vetr.1113

[51] Pun JK. An integrated review of the role of communication in veterinary clinical practice. BMC Vet Res. 2020;16(1):1–14. 33076917 10.1186/s12917-020-02558-2 PMC7569566

[52] Gordon S, Gardner D, Weston J, Bolwell C, Benschop J, Parkinson T. Fostering the development of professionalism in veterinary students: challenges and implications for veterinary professionalism curricula. Educ Sci. 2021;11(11):720.

