# Supplementary material for: Pet caregiver burden in South Korea: key influences and the implications for veterinarians
Source: BMC Vet Res. 2025 May 9;21:331. doi: 10.1186/s12917-025-04787-9 (PMC12063424; doi:10.1186/s12917-025-04787-9)
Supplement: Supplementary file 1 — Supplementary Material 1 [file 12917_2025_4787_MOESM1_ESM.pdf]

Supplementary file 1 — Survey questionnaire[#]

Part 1: Participants and Pets, and Attachment to Pets

1-1. What is your age? (open-ended)

1-2. What is your gender?

1) Male
2) Female

1-3. What is your household's monthly net income (before taxes)? (1,000 Won)

1) <3,000
2) 3,000-5,000
3) 5,000-8,000
4) 8,000<

1-4. What is your household type?

1) Single adult
2) Multiple adults
3) Households with children

1-5. What species of pet(s) do you own? Please select all that apply.

1) Dog
2) Cat

1-6. How many pet(s) do you have? (open-ended)

[If you have multiple animals, please answer based on the one you bring to the veterinary clinic most often.]

1-7. How old is the pet? (open-ended)

1-8. What is the current health status of the pet?

1) Healthy status
2) Curable and/or acute status
3) Chronic and/or terminal status

1-9. Are you the primary caregiver in your family who gives the most effort and time caring for your pet?

1) Yes
2) No

1-10. Please check your level of agreement with the following statements about your pet.[1]

	Strongly disagree	Disagree	Agree	Strongly agree
My pet means more to me than any of my friends.	(0)	(1)	(2)	(3)
Quite often I confide in my pet.	(0)	(1)	(2)	(3)
I believe that pets should have the same rights and privileges as family members.	(0)	(1)	(2)	(3)
I believe my pet is my best friend.	(0)	(1)	(2)	(3)
Quite often, my feelings towards people are affected by the way they react to my pet.	(0)	(1)	(2)	(3)
I love my pet because he/she is more loyal to me than most of the people in my life.	(0)	(1)	(2)	(3)
I enjoy showing other people pictures of my pet.	(0)	(1)	(2)	(3)
I think my pet is just a pet.*	(0)	(1)	(2)	(3)
I love my pet because it never judges me.	(0)	(1)	(2)	(3)
My pet knows when I'm feeling bad.	(0)	(1)	(2)	(3)
I often talk to other people about my pet.	(0)	(1)	(2)	(3)

My pet understands me.	(0)	(1)	(2)	(3)
I believe that loving my pet helps me stay healthy.	(0)	(1)	(2)	(3)
Pets deserve as much respect as humans do.	(0)	(1)	(2)	(3)
My pet and I have a very close relationship.	(0)	(1)	(2)	(3)
I would do almost anything to take care of my pet.	(0)	(1)	(2)	(3)
I play with my pet quite often.	(0)	(1)	(2)	(3)
I consider my pet to be a great companion.	(0)	(1)	(2)	(3)
My pet makes me feel happy.	(0)	(1)	(2)	(3)
I feel that my pet is a part of my family.	(0)	(1)	(2)	(3)
I am not very attached to my pet.*	(0)	(1)	(2)	(3)
Owning a pet adds to my happiness.	(0)	(1)	(2)	(3)
I consider my pet to be a friend.	(0)	(1)	(2)	(3)

*reverse score (for two items)

Part 2: Support for Pet Care

2-1. Do you feel that your family or acquaintances understand and empathize with your worries and challenges related to caring for your pet?

1) Never (0)
2) Rarely (1)
3) Often (2)
4) Always (3)

2-2. Do you receive financial support from family or acquaintances for your pet caring?

1) Never (0)
2) Rarely (1)
3) Often (2)
4) Always (3)

2-3. Do you receive practical assistance from family or acquaintances, such as shared caregiving or help you out (e.g., at work, traveling, or on a business trip)?

1) Never (0)
2) Rarely (1)
3) Often (2)
4) Always (3)

Part 3: Veterinary Services

3-1. How many times have you visited a veterinary clinic for your pet in the past year (from November 2021 to the present)? (open-ended)

3-2. How much have you spent on your pet's veterinary medical care in the past year (from November 2021 to the present)? (open-ended)

3-3. How burdensome do you perceive the veterinary fees for caring your pet?

1) Not burdensome at all (1)
2) Slightly burdensome (2)
3) Moderately burdensome (3)
4) Somewhat burdensome (4)
5) Very burdensome (5)
6) No experience with veterinary clinic visits (excluded)

3-4. Have you ever discussed home care (such as medication, daily care, family support, and other challenges) with a veterinarian during veterinary consultations?

1) Never (0)
2) Sometimes (1)
3) Every visit (2)
4) Often via call/email/messenger (3)

3-5. Have you ever discussed the changing care needs throughout your pet's life-cycle (such as life-stage transitions, age-related illness, and death) with a veterinarian during veterinary consultations?

1) Never (0)
2) Sometimes (1)
3) Every visit (2)
4) Often via call/email/messenger (3)

Part 4: Caregiver Burden

4. Please check your level of agreement with the following restrictions on caring for your pet.[2]

	Never	Rarely	Sometimes	Frequently	Nearly always
Do you feel that because of the time you spend with your pet that you don't have enough time for yourself?	(0)	(1)	(2)	(3)	(4)
Do you feel stressed between caring for your pet and trying to meet other responsibilities for your family or work?	(0)	(1)	(2)	(3)	(4)
Do you feel you have lost control of your life since your pet' s illness?	(0)	(1)	(2)	(3)	(4)
Do you feel angry when you are around your pet?	(0)	(1)	(2)	(3)	(4)
Do you feel embarrassed over your pet's behavior?	(0)	(1)	(2)	(3)	(4)
Do you feel you should be doing more for your pet?	(0)	(1)	(2)	(3)	(4)
Do you feel you could do a better job in caring for your pet?	(0)	(1)	(2)	(3)	(4)

#. This file includes only the questions relevant to this study; questions from other sections of the survey, which are analyzed in separate research papers, are excluded.

1. **the Lexington Attachment to Pets Scale (LAPS)** from Johnson TP, Garrity TF, Stallones L. Psychometric evaluation of the Lexington attachment to pets scale (LAPS). Anthrozoös. 1992;5(3):160-75.

2. **the 7-item abbreviated version of the Zarit Burden Interview (ZBI)** from Spitznagel MB, Mueller MK, Fraychak T, Hoffman AM, Carlson MD. Validation of an abbreviated instrument to assess veterinary client caregiver burden. J Vet Intern Med. 2019;33(3):1251-9.; Leonardi AJ, Fulkerson CM, Shields CG, Childress MO. Veterinary oncologists and pet owners differ in their perceptions of chemotherapy-related adverse events in cancer-bearing dogs. J Am Vet Med Assoc. 2024;262(3):334-42.
